# Electrochemical Etching for Seamless Micro-Transfer Printing of InGaN LEDs

**DOI:** 10.1021/acsaelm.5c00259

**Published:** 2025-05-20

**Authors:** Mikołaj Chlipała, Konstantinos Akritidis, Iryna Levchenko, Krzysztof Gibasiewicz, Tara Brstilo, Maximilien Billet, Pol Van Dorpe, Natalia Fiuczek, Marta Sawicka, Bart Kuyken, Henryk Turski

**Affiliations:** † 243577 Institute of High Pressure Physics Polish Academy of Sciences, PAS, 01-142 Warsaw, Poland; ‡ Photonics Research Group, INTEC Department, 26656 Ghent University—Imec, Technologiepark-Zwijnaarde 126, 9052 Ghent, Belgium; § IMEC, Kapeldreef 75, 3001 Leuven, Belgium; ∥ School of Electrical and Computer Engineering, Cornell University, Ithaca, New York 14853, United States

**Keywords:** gallium nitride, light-emitting diodes, micro-transfer printing, molecular beam epitaxy, electrochemical etching, thin-film structures, heterogeneous integration, optoelectronic devices

## Abstract

The development of complex optoelectronic devices often necessitates efficient and high-quality visible light sources. The gallium nitride (GaN) material family, widely used in constructing light-emitting diodes for general lighting, is an obvious choice for this purpose, but the highest quality devices need to be obtained on native substrates. In this study, we demonstrate the fabrication of LEDs on bulk GaN substrates, which are compatible with microtransfer printing (μTP) technology, enabling integration onto foreign wafers. The structures are grown on a heavily doped n-type sacrificial underlayer realized through plasma-assisted molecular beam epitaxy. Fully processed LEDs are undercut by using electrochemical etching to selectively remove the underlayer, resulting in a thin-film structure with a smooth bottom surface. This smooth surface facilitates the easy integration with foreign wafers. A successful transfer using a micromanipulator and μTP setup was conducted, showing an electrical performance similar to that of the original devices. This work underscores the potential of GaN-based light emitters for advanced optoelectronic applications in integrated circuits and highlights the role that μTP plays in achieving heterogeneous integration.

## Graphical abstract

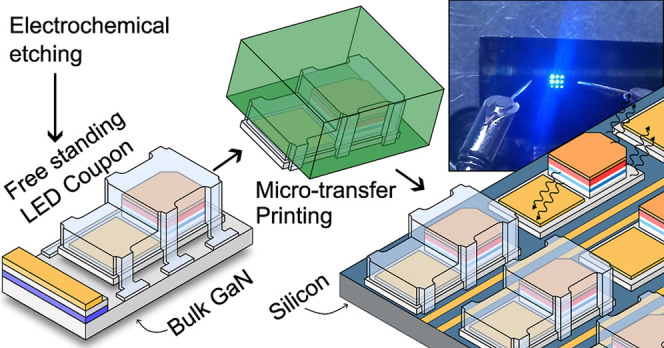

## 1 Introduction

The integration of gallium nitride (GaN)-based structures into more complex optoelectronic systems offers new functionalities,^1^ enabling applications, such as high resolution^2^ or flexible displays,^3,4^ high-performance sensors, and power electronics.^5^ Traditional growth and processing methods for GaN structures, while robust, face limitations in achieving heterogeneous integration across dissimilar, particularly noncrystalline substrates. Microtransfer printing (μTP) has emerged as a versatile solution to these challenges, facilitating the precise assembly of GaN-based microstructures onto various substrates with a high spatial accuracy. This technique involves the transfer of prefabricated microdevices from their source wafers to target substrates, allowing for the combination of materials with differing properties.^6^

μTP requires, in the first step, the delamination of prefabricated structures from their source substrates. This process is referred to in the literature as epitaxial lift-off (ELO) and it can be realized in several different approaches that have been recently reviewed for III-nitride materials and devices, in particular in light of microLED displays.^7,8^ One of the most exploited techniques is laser lift-off (LLO), in which a high-power laser passes through the transparent sapphire and upon light absorption at the interface with GaN, local heating and interface decomposition can occur to enable substrate removal.^9–13^ The careful optimization of the LLO process parameters is required to control the interface roughness^11,14^ and avoid issues with cracking, buckling, thermal shock, etc. Despite several advantages of LLO, such as high speed, area-selectivity, and high yield, it cannot be easily adopted to the structures and devices grown on native bulk GaN substrates due to the absorption of the laser wavelengths in the substrate.

The second widely used method is chemical lift-off (CLO) using a release layer that can be selectively wet etched,^15–17^ resulting typically in higher backside smoothness than LLO.^8^ CLO has been widely investigated and successfully applied for III–V materials, such as GaAs and InP, for which AlAs^18^ and InGaAs^19^ layers, respectively, are sacrificially removed. Due to the high selectivity between nitrides and silicon, releasing thin films of III-nitride heterostructures grown on Si can be performed easily using wet etching, for example, in HF solution leaving GaN intact.^20^ However, for other substrates, e.g., sapphire, SiC, and native GaN, selective chemical etching is difficult to achieve.^21^ CLO techniques for GaN have utilized release layers based on ZnO,^22,23^ CrN,^24^ Nb_2_N,^25^ and other such as SiO_2_ strips.^26^ The gentle removal of a sacrificial layer without thermal stress can be considered advantageous for CLO, but its production efficiency may require solutions to speed up the process, e.g., by patterning the sacrificial layer.^27^ Additional benefits can be achieved by photoelectrochemical etching (PEC)^28,29^ and electrochemical etching (ECE).^30–32^ Then, electron–hole pairs are generated by either UV illumination or an external bias, enhancing oxidation and dissolution of a sacrificial layer.

The third concept enabling the transfer of nitride structures is remote epitaxy or van der Waals epitaxy on graphene,^33,34^ h-BN,^35^ or other 2D materials. It is very appealing and paves the way to the heterogeneous integration of highly mismatched material systems. Moreover, the released epilayer can be transferred onto an arbitrary substrate, and the host substrate can be reused after exfoliation, reducing the overall manufacturing overall cost. However, there are still some challenges to address such as the availability of suitable 2D material substrates in large areas, the fabrication of devices on large-area wafers without material delamination at the weak 2D layer interface, a reliable large-area exfoliation method, and the development of a crack-free transfer process.^36^ The question about the defect density in the nitride layer grown on a 2D material remains open.

Knowing the characteristics of the epitaxial lift-off techniques, the highest backside surface quality, high selectivity, and uniformity of the device separation process can be obtained by ECE. Importantly, it emerges as a viable route to obtain the selective removal of highly n-type doped layers and separate devices from the native bulk substrate.^30–32^ Full removal of a sacrificial layer can be achieved by choosing an appropriately high doping level of a sacrificial layer and optimum etching bias for a given electrolyte.^37–39^ The main challenges are (1) obtaining a smooth N-polar surface free of residuals^39,40^ and (2) etching only the release layer.^41^ The latter requires efficient device structure protection, separating both the device sidewalls and metal layers from being in contact with the electrolyte,^42^ and also avoid parasitic etching through the dislocations.^43^ GaN membranes and flip-chip device architectures were demonstrated without parasitic etching.^42^ Previously reported conditions for electrochemical or photoelectrochemical lift-off did not result in the sufficient backside quality to be compatible with μTP.^44^

This work focuses on the preparation of GaN-based structures for μTP, utilizing ECE for delamination.^45^ By leveraging this approach, we aim to advance the integration of GaN structures epitaxially grown on bulk GaN substrates, which offer the highest crystalline quality, into next-generation optoelectronic systems.

## 2 Experimental Section

The epitaxial stacks were fabricated using plasma-assisted molecular beam epitaxy (PAMBE) on a bulk GaN substrate with threading dislocation densities at a level of 10^4^ cm^2^.^46^ Details on thicknesses and chemical compositions for all layers can be found in Table 1. The key point of the growth process is the heavily doped release layer, comprising a 260 nm-thick In_0.03_Ga_0.97_N:Ge layer doped with Ge at a concentration of 2 × 10^20^ atoms/cm^3^, followed by a 40 nm thick layer doped at 6 × 10^20^ atoms/cm^3^. While these thicknesses and concentrations could be further adjusted, the thicker InGaN layer accumulates additional stress within the structure, which can be detrimental for longer-wavelength emitters requiring high In-content active regions. InGaN:Ge was used as it allows for the highest carrier concentration without significantly deteriorating surface morphology.^47^ Growth itself of such heavily doped layers could pose significant challenges in temperature stability,^48^ which we address by frequent and periodic growth interruptions every 20 nm. During each step, we monitor the metal desorption time to ensure a similar average growth temperature during the period. The release layer growth was followed by a 1 μm GaN:Si layer doped at 2 × 10^18^ atoms/cm^3^. The relatively low doping concentration used here ensures that the layer remains unaffected by ECE, while simultaneously providing adequate contact resistivity and current spreading in the lateral injection. A single quantum well (QW) LED structure was subsequently grown following conditions described in ref 49. The layer thicknesses compositions doping and unintentional doping (UID) densities are indicated in Table 1.

**Table 1:** Layers, Doping, and Composition of LED with the Release Layer

			doping	
Function	thickness (nm)	material	element	density (atom/cm^3^)
p-type	5	In_0.14_Ga_0.86_N	Mg	6 × 10^20^
	40	In_0.02_Ga_0.98_N	Mg	3 × 10^19^
	150	GaN	Mg	7 × 10^18^
	20	Al_0.13_Ga_0.87_N	Mg	3 × 10^19^
active region	20	In_0.03_Ga_0.97_N	UID	5 × 10^16^
	2.6	In_0.22_Ga_0.78_N		
	0.5	In_0.17_Ga_0.83_N		
	30	In_0.03_Ga_0.97_N		
n-type	1000	GaN	Si	5 × 10^18^
release layer	40	In_0.03_Ga_0.97_N	Ge	6 × 10^20^
	260	In_0.03_Ga_0.97_N	Ge	2 × 10^20^
buffer	100	GaN	Si	5 × 10^18^
substrate		GaN	O	1 × 10^19^

The proposed process flow for the preparation of the μTP-ready LED coupons is schematically illustrated in Figure 1. After the epitaxial growth shown in Figure 1a, LED processing is performed (see Figure 1b). The samples were first cleaned, and a Ni/Au/Pt (25/75/60 nm) contact to p-type layers was blanket deposited. Photolithography was then performed to define nearly rectangular p-contacts of the size 50 × 50 μm^2^. In the subsequent reactive ion etching (RIE) process, both the metallization using Ar and the LED mesa using Ar and Cl mixtures were etched, with the etching depth reaching the top of the 1000 nm GaN:Si cap layer. After the process, chlorine was removed from the water. This step concluded with a rapid thermal annealing process at 500 °C for 10 min in an N_2_ and O_2_ mixture (7:3). Then, second photolithography was used to define the placement of the Ti/Al/Ni/Au (30/60/40/75 nm) contact pad using the lift-off process. Contact was positioned 5 μm away from the LED mesa on the GaN:Si layer. The same metal stack, which is placed on the edge of the wafer, is used to bias the sample during the ECE process. The third step involved photolithography to define device separation and coupon mesa. RIE was used to etch through all of the epitaxial layers, reaching the substrate and exposing the sidewalls of the release layer. The sample at this stage is illustrated in Figure 1b. Now, a dielectric stack of 100 nm SiO_2_/1000 nm SiN/100 nm SiO_2_ was blanket deposited. Photolithography and RIE utilizing SF_6_ were then applied to shape the dielectric into designed tethers while also fully covering the metal contacts (Figure 1c). In the final photolithography step, a thick photoresist was used to cover two neighboring coupons and leave only one side of the release layer exposed (Figure 1d). This restricted the ECE from proceeding in only one direction for each coupon. As an electrolyte in the process, we used oxalic acid, which has low toxicity and is biodegradable.^50^ Etching was conducted in a standard three-electrode setup with the whole sample submerged in a 0.3 M solution at the 3 V bias applied to the anode for 8 h (see Figure 1e).^38^ Etching time was chosen based on confirmation that it results in the full release of the structure, rather than optimizing for the shortest process, which could be imperative for future applications. The n-type electrode outside the LED coupons was not submerged in the electrolyte, and the sacrificial layer underneath remained unetched. After the ECE process, the photoresist was removed using an oxygen plasma, and the LED coupons were held in place only by the SiO_2_/SiN/SiO_2_ protective cover, as illustrated in Figure 1f. In Figure 2, LED coupons after ECE are presented. Optical microscopy (Figure 2a) and scanning electron microscopy (SEM) (Figure 2b) are compared. To clearly indicate electropolishing (complete removal of the release layer) in the ECE process, a larger magnification SEM image is presented in Figure 2c. The optimized procedure of ECE allows the selective removal of the sacrificial layer without affecting the coupon facets.

**Figure 1:**
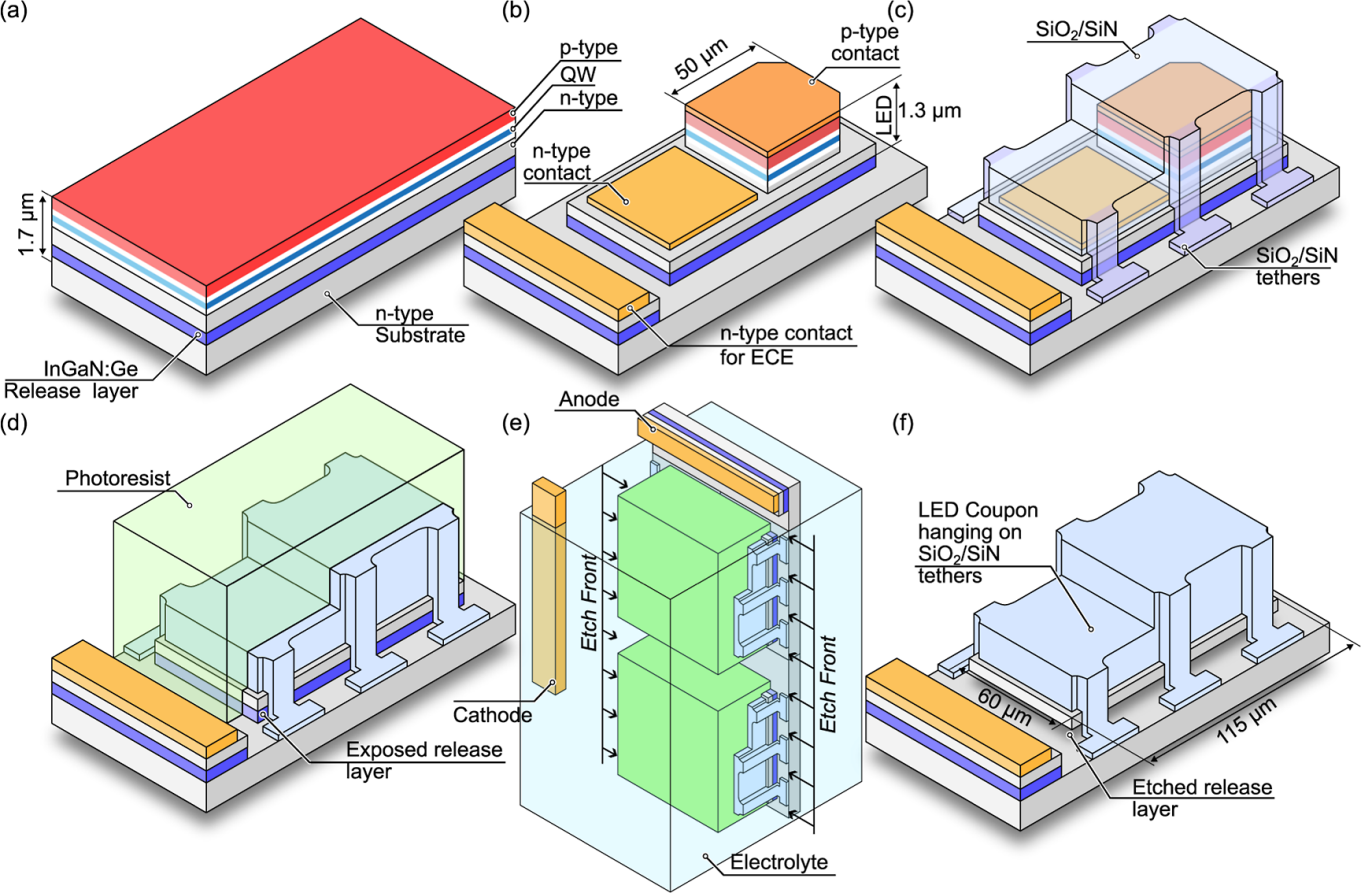
Simplified process flow illustrating (a) epitaxial growth, (b) LED processing with an extra metal contact on a side for ECE, p-type contact consist of Ni/Au/Pt (25/75/60 nm) stack and n-type contact of Ti/Al/Ni/Au (30/60/40/75 nm), (c) device encapsulation, (d) protective photoresist deposition, (e) electrochemical etching of entire wafer submerged in oxalic acid, and (f) photoresist removal resulting in suspended and encapsulated LED coupons ready for transfer onto foreign substrates.

**Figure 2:**
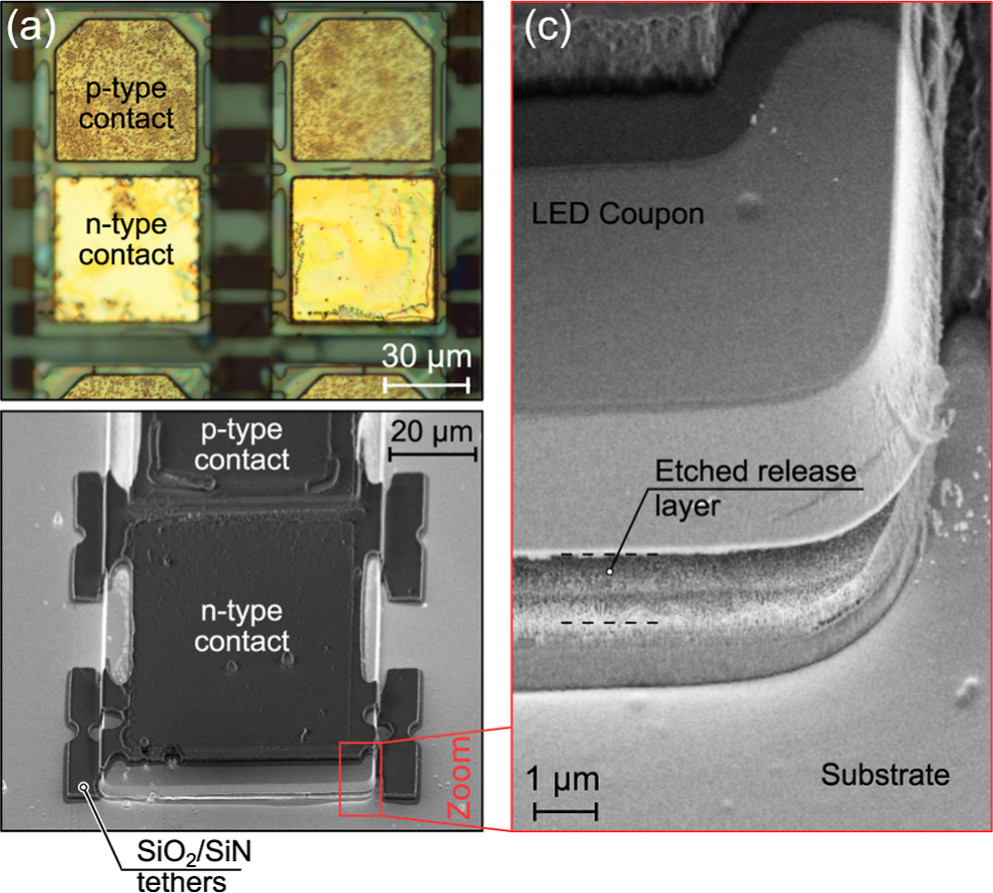
LED coupon with an etched release layer covered by SiO_2_/SiN/SiO_2_ as obtained by (a) optical microscopy and (b) scanning electron microscopy. In (c) zoomed-in part of the coupon indicating separation from the substrate is shown.

## 3 Results and Discussion

The crucial aspect of the preparation of coupons for heterointegration is the flatness of the coupon after the etching process.^29,51^ Figure 3a,b shows atomic force microscopy (AFM) images measured on 10 × 10 and 2 × 2 μm^2^ area, respectively, for the InGaN:Ge/GaN:Si release layer grown on a calibration sample. Figure 3c shows the respective sample schematics. Figure 3d,e presents AFM images of the backside of an LED coupon after ECE delamination from the substrate by using adhesive tape. The respective sample schematic is shown in Figure 3f. The calibration sample shown in Figure 3a–c is grown using identical growth conditions as the release layer grown under LED. The root mean square (RMS) roughness of the release layer after epitaxy is 0.6 nm, measured on both 2 × 2 μm^2^ and 10 × 10 μm^2^ (Figure 3a,b, respectively). The RMS roughness on the backside of an LED is 0.6 nm; see Figure 3d. Note that using a low voltage of 3 V, we were able to obtain coupons with an extremely flat bottom surface, comparable with the roughness of the release layer. Pronounced surface undulations seen in the images in Figure 3a,d support that the obtained backside LED surface roughness is limited by the release layer morphology rather than the ECE procedure. The RMS roughness on a 2 × 2 μm^2^ area of the backside of the coupon presented in Figure 3e is 1.2 nm, which is likely dominated by smaller residues present at the coupon. Further optimization of the growth conditions of the release layer could be promising to improve the smoothness to even lower values. Obtaining such high doping levels as *n* = 6 × 10^20^ cm^–3^ up to now has only been possible for GaN:Ge in PAMBE. Silicon doping both in MBE and MOVPE causes a significant decrease in structural quality above 1 × 10^19^ cm^–3^ ^52^ and 5 × 10^19^ cm^–3^,^53^ respectively. Increasing sacrificial layer doping has been reported to improve the material removal efficiency after ECE in the nitric acid–based electrolyte, leading to the GaN membrane N-polar surface roughness decrease from 8 to 5 nm when doping increased from *n* = 1 × 10^19^ cm^–3^ to *n* = 5 × 10^19^ cm^–3^ and an etching bias was decreased from 17 to 6 V.^39^ Further improvement was done by increasing the polarization field, and the RMS roughness of the backside of the GaN membrane was 0.44 nm as measured on 5 × 5 μm^2^.^39^ This concept of polarization-aided ECE implemented to separate a fully processed device structure, i.e., a resonant-cavity UV LED, resulted in 1.5 nm RMS roughness of the N-polar side of AlGaN layer.^42^ In our case, the GaN coupon backside RMS roughness reaching 0.6 nm was obtained thanks to a (i) high-quality epitaxial interface of initially low roughness and (ii) extremely high doping of InGaN:Ge *n* = 6 × 10^20^ cm^–3^ of the topmost sacrificial layer.

**Figure 3:**
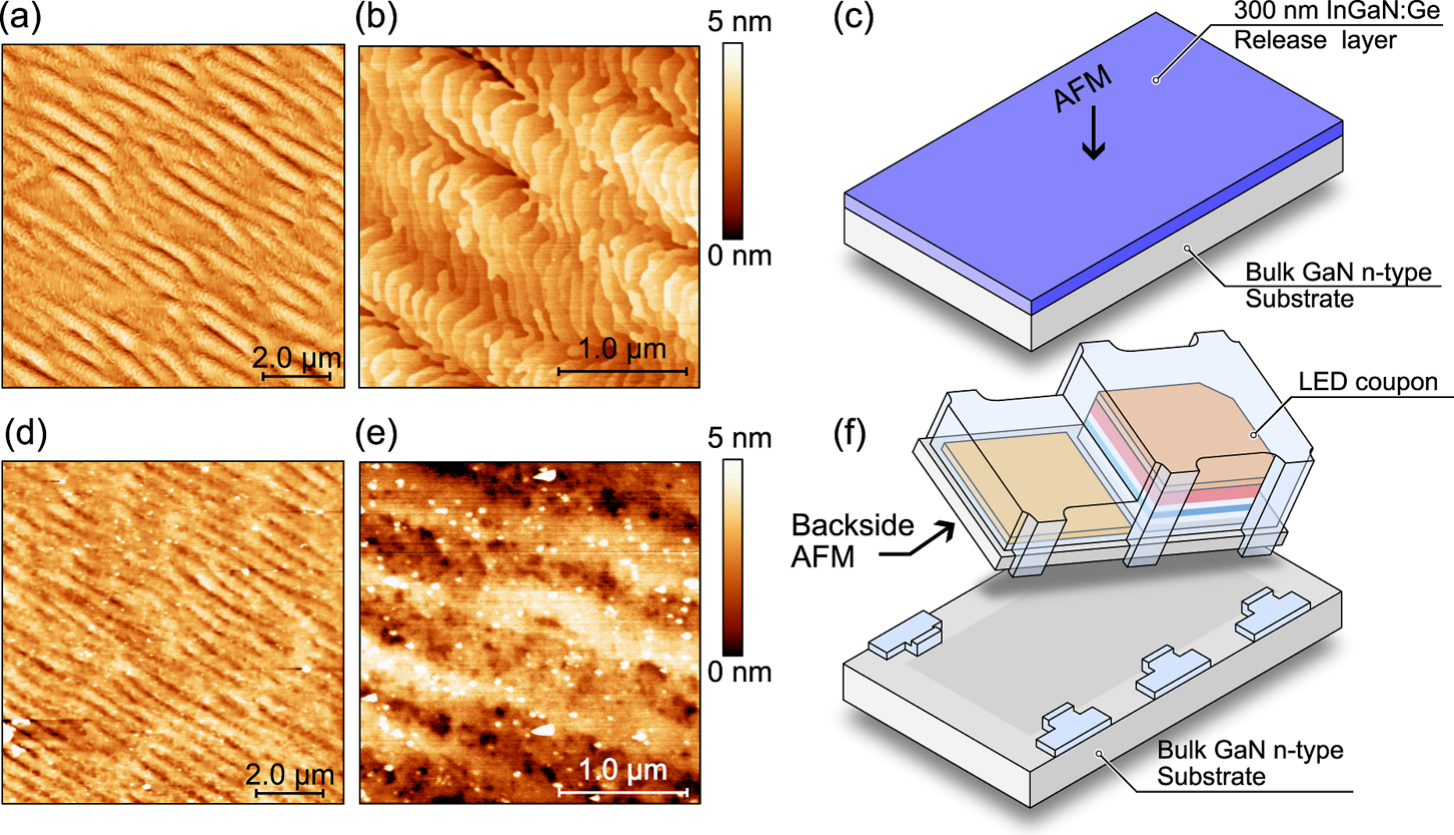
Atomic force microscopy images of (a,b) surface of InGaN:Ge release layer taken on a calibration sample and (c) corresponding sample schematics, and (d,e) surface morphology of the backside of an LED coupon after removal of the release layer by ECE with (f) the corresponding sample schematics.

To perform a straightforward test of the robustness of the LEDs after the ECE process, the whole sample was subjected to SF_6_, exposing the metal contacts and simultaneously thinning the holding tethers (Figure 4a). An individual LED coupon was transferred from the source GaN wafer with many LEDs (Figure 4b) to a dummy Al_2_O_3_ carrier wafer (Figure 4c) by using a micromanipulator equipped with a probe. After the procedure, when forward biased, a clear sign of the visible light emission could be resolved. Current density versus voltage (*j*–*V*) characteristics of the coupon are shown in Figure 5a. The device exhibits comparable *j*–*V* characteristics before and after the transfer, with only a slight decrease in the on-current density observed post-transfer. This difference could also be related to the incomplete removal of the dielectric from the device, resulting in contact resistance dependent on the placement of the probe. For the setup used, only a limited amount of light could be collected. We characterized the optical performance by using an optical fiber to measure power and emission spectra. In Figure 5b, the optical power and external quantum efficiency (EQE) as a function of current density are compared for the same coupon before and after transfer. EQE is presented in a normalized form by dividing by the maximum value of the curve after transfer. Keeping in mind the unoptimized collection of light and the fact that the EQE maximum remains at the same current, this indicates a negligible deterioration of the coupon during transfer.^54^ The emission spectra for different injected currents after transfer are shown in Figure 5c. At low current densities, the LED emits green light, which blue-shifts toward cyan as the current increases (visible also for true-color images presented as insets). This behavior is well described for III-nitride QWs and is caused dominantly by the screening of the built-in polarization field, which leads to a reduced quantum confined Stark effect.^55^

**Figure 4:**
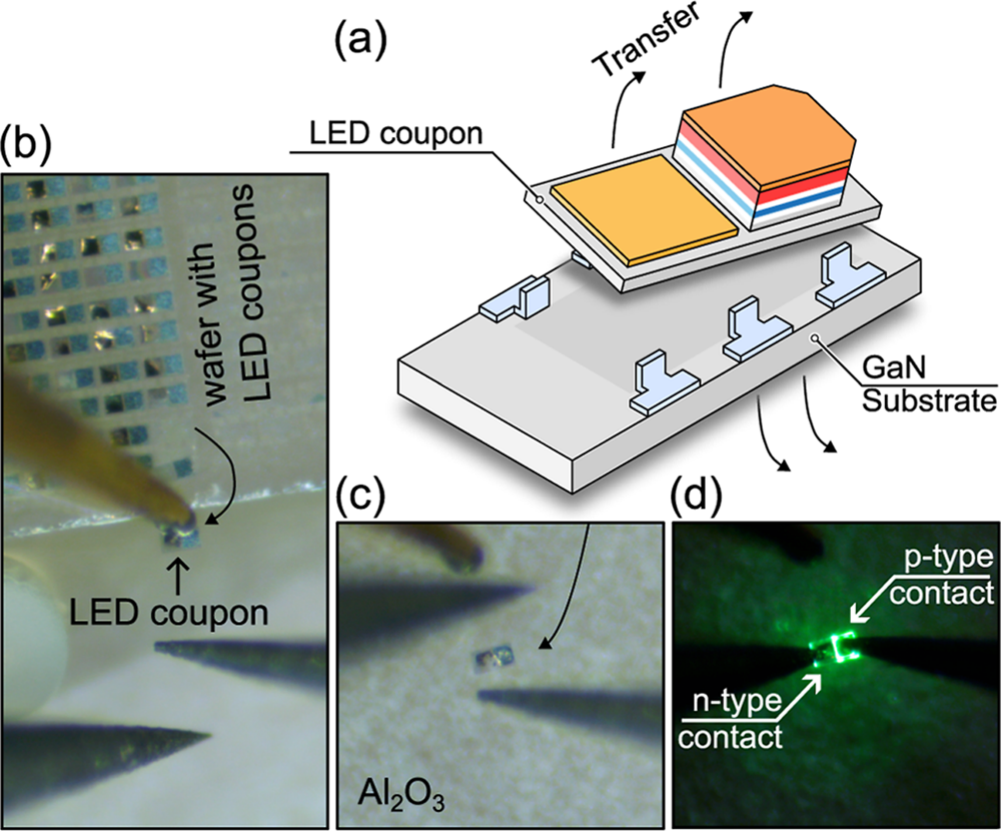
(a) Released LED coupon after ECE. Optical microscopy images of the ultrathin LED (b) stuck to transfer-probe, (c) transferred onto Al_2_O_3_ wafer, and (d) under a forward bias, post-transfer.

**Figure 5:**
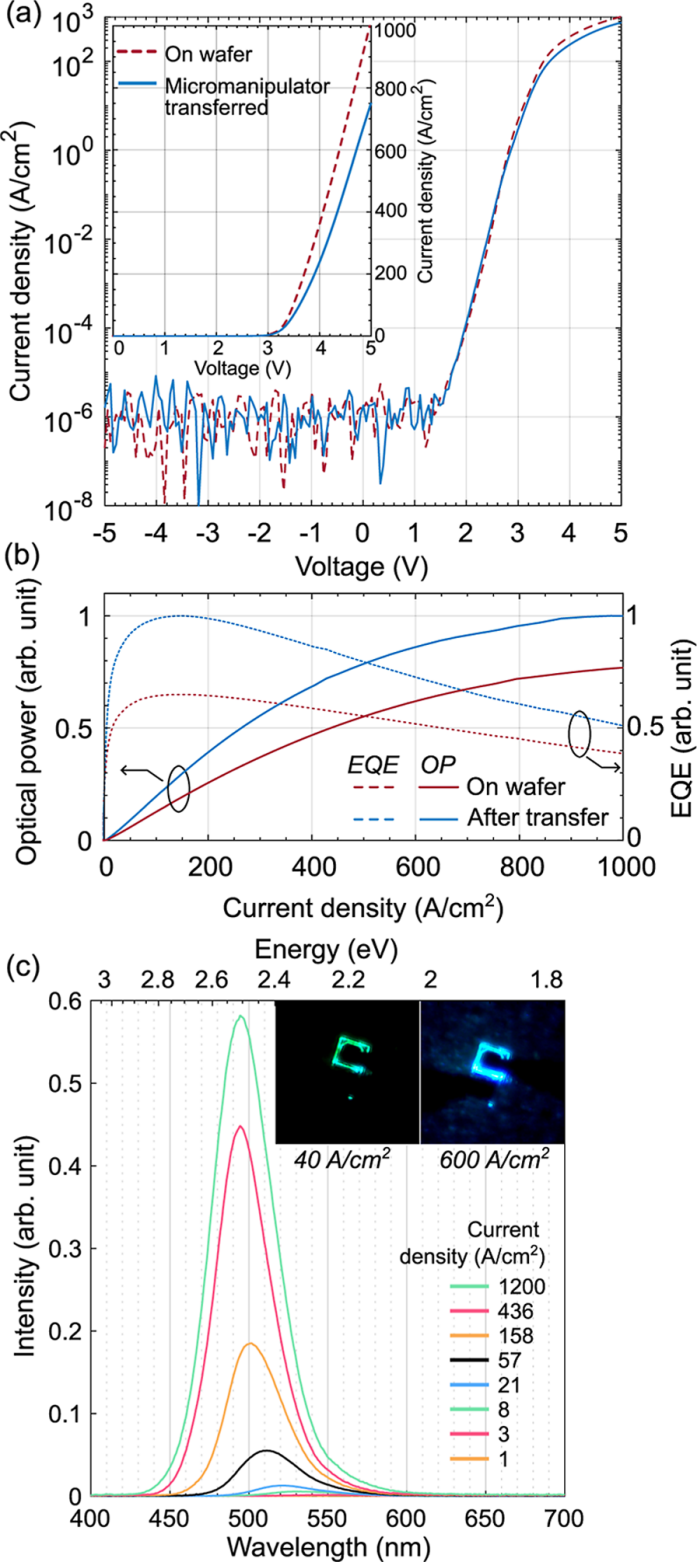
Characteristics for the ultrathin LED obtained before and after transfer. (a) Current density vs voltage in semilog and linear scale and (b) optical output power and external quantum efficiency normalized by dividing by the maximum value of the curve after transfer vs current density. (c) Emission spectra for the device after transfer. The inset presents optical microscopy photos of the device under a forward current.

Source wafer with devices covered by dielectric and after ECE (at the stage presented in Figure 2) was shipped to Ghent University-imec for μTP tests. This transfer procedure was carried out according to the scheme presented in Figure 6. The free-standing LEDs were picked, breaking the tethers, from the source wafer by using a polydimethylsiloxane (PDMS) stamp, Figure 6b. An optical microscopy image of the topography of the bottom surface of the picked coupons can be seen in Figure 6c. Subsequently, the LEDs were transferred onto a silicon substrate, which was spin-coated prior to a 40 nm adhesive divinylsiloxane-bis-benzocyclobutene (DVS-BCB, or BCB) layer. The printing (Figure 6d) took place at an elevated temperature of 80 °C. With multiple LED coupons integrated onto the target wafer (Figure 6e), BCB curing was performed at 270 °C. An exemplary optical microscopy image of six LED coupons after subsequent transfer procedures and curing is presented in Figure 6f. The presence of dielectric encapsulation at this point is confirmed by a few tethers that were transferred together with the coupon. To electrically characterize devices at this point, the SF_6_ blanket etching was used to remove the SiO_2_/SiN/SiO_2_ dielectric. Microtransfer-printed coupons from the same process characterized in Figure 5 exhibit similar *I*–*V* characteristics, as confirmed by exemplary *j*–*V* characteristics for 11 coupons presented in Figure 7. Nine LED coupons, each of the same size as before, were also transferred to a separate wafer, where after dielectric removal, 350 nm thick SiN was deposited for device isolation using plasma-enhanced chemical vapor deposition. Then, openings in that dielectric were etched in the middle of each LED contact using SF_6_ and additional metallization (40 nm/1 μm, Ti/Au) was deposited to connect LEDs into a parallel circuit. As shown in the inset of Figure 7, under the same bias, they emitted light at comparable intensities with some random variations also visible in j-V characteristics measured for separated devices presented in Figure 7.

**Figure 6:**
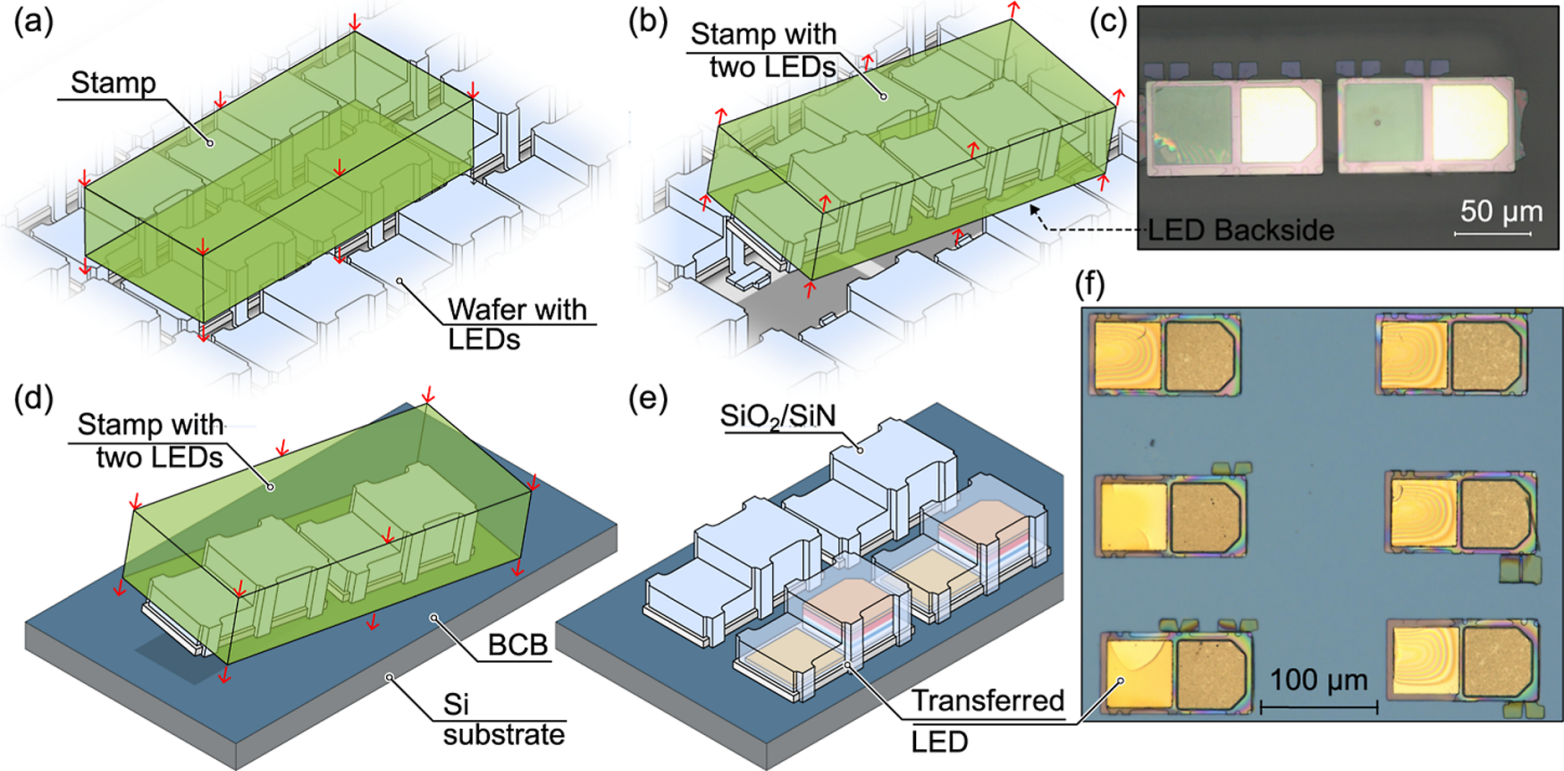
Scheme showing microtransfer printing procedure. (a,b) Coupon pick-up procedure is shown, while (c) a corresponding optical microscopy image of LED coupons on a transfer-printing stamp. The printing procedure (d) with the final stage presenting the scheme and real-life optical microscopy image after transfer (e,f), respectively.

**Figure 7:**
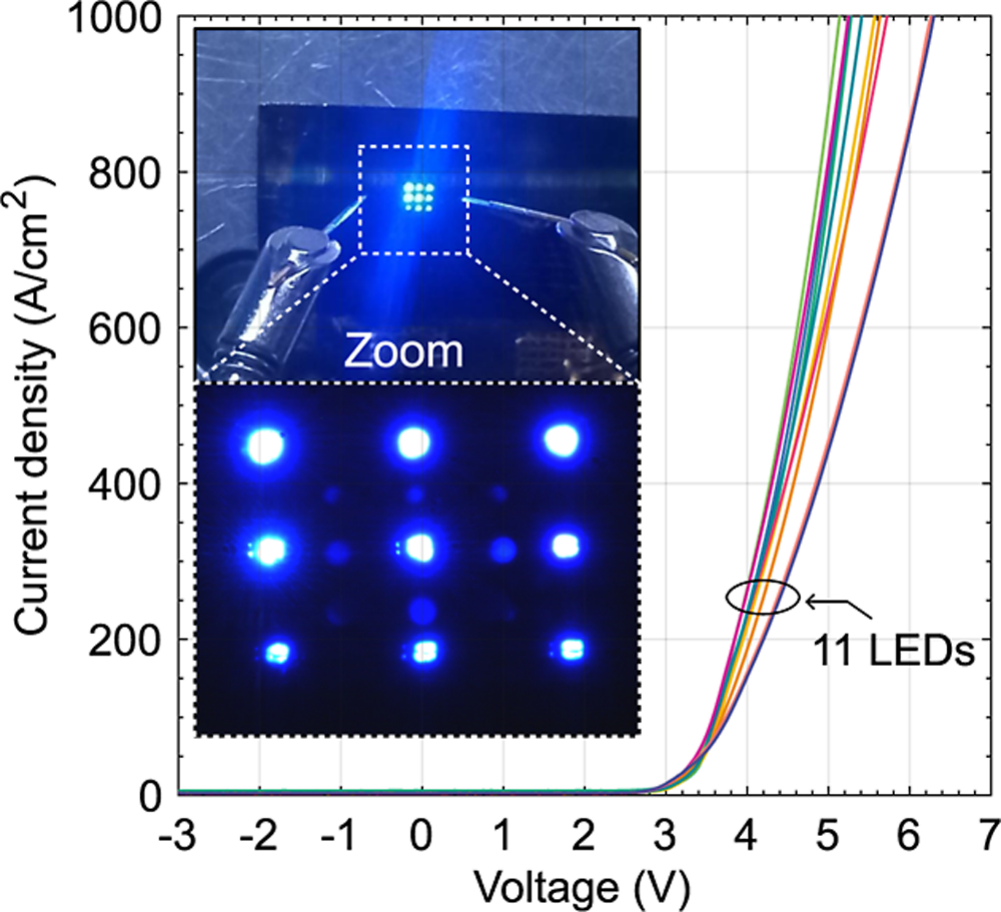
Current density as a function of voltage for 11 coupons after the microtransfer printing procedure is shown in Figure 6. The inset shows the operation of an LED array of nine devices connected in parallel.

## 4 Conclusions

The presented data demonstrate the successful delamination of ultrathin LEDs from bulk GaN substrates. Devices could then be transferred to foreign substrates without significant degradation in performance. ECE has proven to be a reliable technique for producing freestanding μTP-ready thin-film LEDs. By optimizing the ECE conditions, a uniform removal of the release layer was achieved, resulting in an exceptionally smooth backside of the coupons, which ensured excellent adherence to foreign substrates. The dielectric tethers used were stiff enough to keep the coupons in place during sample shipment and thin enough to release the device during transfer.

The use of high-quality bulk GaN substrates enables the creation of structures with threading dislocation densities below 10^5^ cm^–2^ that can be integrated onto foreign substrates, which is important for applications relying on a high current density operation. ECE offers a unique possibility to delaminate such structures. Additionally, because the source wafer is not destroyed in the process, such substrates can be recycled, substantially reducing the costs of their use in production.

The presented approach also has its challenges. The high selectivity of the ECE process is ensured by a large difference in doping concentrations within the structure, which leads to a more demanding and longer epitaxial growth process. Additionally, the more complicated device processing, including the long ECE process, could prove challenging for large-area samples. We believe that further optimization of the ECE conditions, such as the electrolyte concentration and process temperature, should result in a more industrially compatible process flow.

The presented results were obtained for PAMBE-grown structures to ensure high germanium doping in the release layer. Here, for simplicity, the same growth technique was used to realize the LED structure on top of it. In future applications, heavily doped templates prepared by one technique could be used for the subsequent growth of light emitters in a separate process realized by a different growth technique. Alternatively, lower doping concentrations of the release layer could suffice for optimized ECE etching conditions, leading to an even smoother back surface of the transferred devices.

## Data Availability

The data underlying this study are openly available in the Repository for Open Data RepOD at 10.18150/KGI4NU.
